# The Protecting Activity of RIPACUT^®^: A New Therapeutic Approach Preserving Epithelial Health Based on the Combination of Iceland Lichen Extract, Silver Salt, and Sodium Hyaluronate

**DOI:** 10.3390/life13051088

**Published:** 2023-04-26

**Authors:** Raffaella Belvedere, Nunzia Novizio, Daniela Eletto, Amalia Porta, Umberto Di Maio, Antonello Petrella

**Affiliations:** 1 Department of Pharmacy, University of Salerno, Via Giovanni Paolo II 132, 84084 Fisciano, Italy; 2 Shedir Pharma Group Spa, Via Bagnulo 95, 80063 Piano di Sorrento, Italy

**Keywords:** RIPACUT^®^, sodium hyaluronate, silver nitrate, Iceland lichen extract, oxidation, anti-inflammatory activity, antimicrobial effects

## Abstract

Epithelial integrity and function must be maintained in a dynamic healthy equilibrium, keeping unaltered the oxidative and inflammatory conditions and the microbiome of the cutaneous layers. Beside the skin, other mucous membranes can be injured, such as the nasal and anal ones, because of the contact with the external environment. Here, we detected the effects of RIPACUT^®^, a combination of Iceland lichen extract, silver salt and sodium hyaluronate that individually act in diverse biological ways. The findings we obtained on keratinocytes, nasal and intestinal epithelial cells reveal that this combination showed a marked antioxidant activity, further assessed by the DPPH assay. Additionally, by analyzing the release of the IL-1β, TNF-α and IL-6 cytokines, we proved the anti-inflammatory effect of RIPACUT^®^. In both cases, the main preserving action was due to Iceland lichen. We also observed a notable antimicrobial activity mediated by the silver compound. These data suggest that RIPACUT^®^ could signify the basis for an attractive pharmacological approach to maintaining healthy epithelial conditions. Interestingly, this may be extended to the nasal and anal areas where it protects against oxidative, inflammatory and infectious insults. Thus, these outcomes encourage the creation of sprays or creams for which sodium hyaluronate can guarantee a surface film-forming effect.

## 1. Introduction

Good skin care practices involve specific choices primarily focused on the maintenance of a healthy lifestyle and using topical products that improve the quality of epithelial layers. It is important to control the delicate balance between oxidative stress and antioxidants, prevent superficial infections and contain inflammatory reactions [1]. With regard to the antioxidant aim, this approach generally provides the use of natural products, among which, in recent years, Iceland lichen has attracted great attention. The antioxidant properties of its extract have already been proven by using chemical agents [2]. In addition to these findings, the immunological effects attributable to Iceland lichen have been described, partially explaining its use in popular medicine for years for the treatment of several inflammatory conditions [3]. Among the physiological and healthcare applications, we find the emollient and soothing action (oropharyngeal mucosa), digestive function, promotion of fluidity of bronchial secretions [3]. Moreover, the management of physical epithelial barriers becomes necessary after several injuries such as trauma, radiotherapy, chronic infection or surgery. In this scenario, hyaluronic acid has been described in depth thanks to its cytoprotective action in cutaneous and mucous surfaces. Indeed, the hyaluronic acid’s structure gives it unique physiochemical and biological properties that depend on molecular weight. Following tissue damage, high-molecular-weight hyaluronic acid undergoes degradation; for this reason, exogenous administration of this compound is often considered a useful strategy to keep these body barriers intact [4,5]. Therefore, the healthy conditions of the epithelium can also be assessed based on the presence of microorganisms. The airway, anal and cutaneous microbiomes have to be maintained stably balanced [6]. For that reason, research efforts have been made to refine advanced strategies based on simple antimicrobial agents such as silver compounds. The substantial clinical use of various silver-based formulations is due to their high biological activity against bacteria and fungi, but also due to their little toxicity in the human body and the minimal risks associated to exposure by ingestion, inhalation, dermal, urological or hematogenous application [7]. In fact, in addition to the skin, it is important to preserve the integrity of several internal mucosal membranes such as the nasal or anal ones, which are known to be more sensitive due to their contact with the external environment.

In all cases, the use of specific topical creams or sprays could induce a dermal protective effect against pain during or after an examination, such as a biopsy, in the presence of neoplastic or inflammatory diseases, in case of superficial abrasions, to facilitate the resolution of lesions [8,9]. Thus, it has become important to expand the study of topical pharmacological products for specific epithelial mucosa such as the nasal and anal ones as well. Based on this information, in this study, each molecule, i.e., of Iceland lichen extract, sodium hyaluronate and silver salt, was evaluated as a single component and in different types of mixture prior to the definitive assessment of RIPACUT^®^.

In particular, Iceland lichen has attracted marked attention in recent years as a useful substance for several kinds of biological processes thanks to the different chemical molecules of which it is composed. Interesting antimicrobial, antioxidant, anticancer and genotoxic activities have been revealed for *Cetraria islandica* extract [10]. *Cetraria islandica* Ach. represents the most investigated species from the pharmacological perspective among the 20,000 lichen species described worldwide [11].

Thus, we evaluated the antioxidant and antimicrobial role of RIPACUT^®^ in vitro and its ability to inhibit the release of the main proinflammatory cytokines. Thus, this work intended to formulate valid assumptions for the development of RIPACUT^®^ as a cream or spray for skin, nasal and anal topical formulations with multiple beneficial activities keeping the normal mucosal barrier status in terms of integrity, hydration, presence of microorganisms and redox features. Indeed, all these conditions have to be considered in order to guarantee a healthy status of epithelial layers, but also to facilitate the recovery after different kinds of physiopathological insults.

## 2. Materials and Methods

### 2.1. Cell Lines

Normal human dermal keratinocytes (HNEK) were primary keratinocytes obtained from juvenile foreskin of a pool of donors and were acquired from PromoCell (Atlanta, GA, USA). These cells were grown in a ready-to-use Keratinocytes Growth Medium 2 (PromoCell; Atlanta, GA, USA). Human nasal epithelial cells (HNEpC; PromoCell; Atlanta, GA, USA) were primary cells cultured in a ready-to-use Airway Epithelial Cell Growth Medium (PromoCell; Atlanta, GA, USA). Human colon epithelial cells FHC (ATCC; Manassas, VA, USA) were cultured in DMEM:F12 (ATCC; Manassas, VA, USA) supplemented with 10 mM HEPES; 20 ng/mL human recombinant EGF (Thermo Fisher; Waltham, MA, USA); 10% fetal bovine serum (FBS; Euroclone, Milan, Italy). The cells were stained at 37 °C in a humidified 5% CO_2_ and 95% air atmosphere. The primary cells were maintained in culture until passage 10.

### 2.2. Preparation of Substances Solutions

Sodium hyaluronate (SH) was provided by Lehvoss srl (Origgio, Italy) as a white powder with purity grade ≥ 95.0 and dissolved in deionized sterile water in order to obtain a 2 mg/mL stock solution. Silver nitrate (SN) crystals with purity grade ≥ 99.0 were supplied by Farmalabor srl (Canosa di Puglia, Italy) and dissolved in sterile deionized water to obtain a 100 mg/mL concentration. Moreover, these substances were used as reported in [12]. Finally, finely powdered Iceland lichen extract (IL) with purity grade ≥ 90.0 was provided by Nutraceutica srl (Monterenzio, Italy) and dissolved in DMSO to obtain a 20 mg/mL stock solution. This dry extract was obtained from the thallus of the *Cetraria islandica* Ach. plant by using water as an extraction solvent with the aid of < 0.5% colloidal anhydrous silica and including maltodextrin as an excipient (the loss on drying was evaluated to be < 5%). The particle size was ca. 35 mesh with a density of 0.5 g/mL and the presence of Pd < 3 ppm, Cd < 1 ppm and Hb < 0.1 ppm. Then, these solutions were diluted in a cell growth medium or 1× PBS to obtain the needed final concentrations as follows:

- SH: 200 µg/mL;
- SN: 30 µg/mL;
- IL: 50, 100, 150 and 200 µg/mL; to reach the concentration of 2000 µg/mL on the bacterial medium.

### 2.3. 3-(4,5-Dimethylthiazol-2-yl)-2,5-Diphenyltetrazolium Bromide (MTT) Assay

As described in [13], the HNEK, HNEpC and FHC cells were harvested and cell viability was calculated through the MTT assay. At 24, 48 and 72 h of treatments, 25 μL 5 mg/mL MTT stock solution was added to each well in 100 μL medium for 3 h at 37 °C. Next, the cells were lysed in DMSO (100 μL/well). The optical density (OD) of each well was measured with a microplate spectrophotometer (Titertek Multiskan MCC/340) equipped with a 550 nm filter. The viability of the cells in response to treatment was calculated as follows: % viable cells = (OD of the treated cells/OD of the control) × 100.

### 2.4. 1,1-Diphenyl-2-Picrylhydrazyl (DPPH) Assay

A spectrophotometry technique was performed by using the DPPH scavenging radical assay in order to determine the antioxidant activity. DPPH (0.04 mg/mL; Sigma-Aldrich Co.; St. Louis, MO, USA) was prepared in methanol to reach an absorbance of about 2.5 at 520 nm when measured with a multiwell-reading spectrophotometer (Titertek Multiskan MCC/340). To test the substances, 150 µL of each of them, as single compounds and in combination, were mixed in a 96-well plate with 150 µL of the DPPH solution. After 30 min of incubation at room temperature in the dark, absorbance at 520 nm was used to calculate the radical scavenging activity as the percentage of inhibition, as described in [14]. The values obtained for each positive compound were used to determine the EC_50_ values (the concentration at which 50% of the free radical DPPH is reduced) at each timepoint.

### 2.5. 2′,7′-Dichlorodihydrofluorescein Diacetate (DCHF-DA) Assay

DCFH-DA (Sigma-Aldrich Co.; St. Louis, MO, USA) was used to test the intracellular oxidative stress. Briefly, 1.5 × 10^5^/well HNEK, HNEpC and FHC cells were plated and treated for 24 h at the selected conditions. A pretreatment with 100 µM CoCl_2_ (Sigma-Aldrich Co.; St. Louis, MO, USA) for 24 h was performed as an oxidative stimulus. Next, the cells were further incubated with 10 μM DCHF-DA for 15 min at 37 °C in the dark. The cells were analyzed with a Becton Dickinson FACScan flow cytometer (BD FacsCalibur; Milan, Italy) by means of the Cells Quest program [15]. The fluorescence intensity was calculated on a two-parameter dot plot reporting the side scatter (SSC) on the Y-axis vs. the FL1-H on the X-axis. It was divided into two quadrants by a margin on the second subdecade of the 10^1^ value of the log_10_ scale on the X-axis. These events were calculated as the percentage of the total number of cells visualized on the whole dot plot.

### 2.6. Antimicrobial Activity

The antimicrobial activity of each compound was assessed for the cell growth of *Staphylococcus aureus* (ATCC 6538), *Staphylococcus epidermidis* (ATCC 12228), *Pseudomonas aeruginosa* (ATCC 27853), *Escherichia coli* (ATCC 8739). The test solutions were incubated with each bacterial strain and their inhibitory effect in vitro was determined as reported in [16]. Briefly, the cultures were kept overnight in a Mueller–Hinton agar (Becton Dickinson and Company; Franklin Lakes, NJ, USA), then diluted at 5 × 10^5^ CFU/mL as the final concentration in a Mueller–Hinton broth containing the respective IL, SN and SH solutions for 24 h at 37 °C. The effects of the tested conditions were determined by reading the bacterial cultures at 600 nm and comparing their viability to the non-treated control.

### 2.7. Enzyme-Linked Immunosorbent Assay (ELISA)

After the different kinds of treatments with 200 µg/mL SH, 30 µg/mL SN and 150 µg/mL IL for 24 h, the HNEK, HNepC and FHC supernatants were harvested, and the secreted quantity of interleukin 1β (IL-1β), tumor necrosis factor α (TNF-α) and interleukin 6 (IL-6) was evaluated with a fitting human ELISA kit as per the manufacturer’s instructions (Diaclone; Besançon, France), as reported in [17]. The whole combination was also compared to the inflammatory stimulus of 1 µg/mL LPS (Sigma-Aldrich Co.; St. Louis, MO, USA) incubated with the cells for 24 h.

### 2.8. Statistical Analysis

All the results are the means ± SD (standard deviation) of at least three experiments performed in triplicate, as described in [18]. Statistical comparisons between the groups were made using a two-tailed *t*-test comparing two variables. The differences were considered significant if *p* < 0.05, *p* < 0.01 and *p* < 0.001.

## 3. Results

### 3.1. The Substances We Considered, Alone or Differently Mixed Together, Did Not Affect Cell Viability

**Figure 1:**
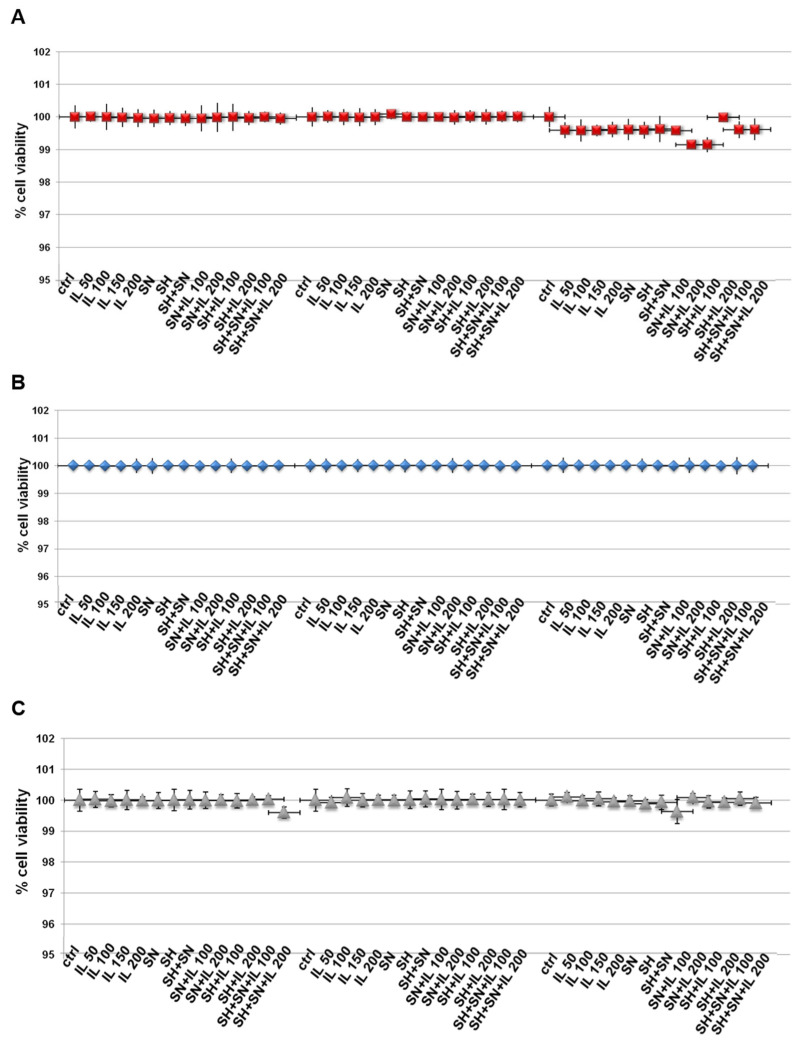
MTT was performed after the HNEK (**A**), HNEpC (**B**) and FHC cells (**C**) were treated for 24, 48 and 72 h with IL (50 µg/mL, 100 µg/mL, 150 µg/mL and 200 µg/mL), SH (200 µg/mL), SN (30 µg/mL), SH + SN, 100 µg/mL IL + SN, 200 µg/mL IL + SN, 100 µg/mL IL + SH, 200 µg/mL IL + SH, 100 µg/mL IL + SH + SN and 200 µg/mL IL + SH + SN. The data are represented as the means ± SD. No differences with statistical significance were found.

In order to evaluate some biological effects of Iceland lichen extract (IL), sodium hyaluronate (SH) and silver nitrate (SN), we initially examined the viability of HNEK, HNEpC and FHC cells treated with these compounds. By means of the MTT assay, we discovered that none of these substances induced any kind of cytotoxic action on all cell lines (Figure 1A–C for HNEK, HNEpC and FHC cell lines, respectively) at 24, 48 and 72 h. SH and SN were tested at the concentrations of 200 µg/mL and 30 µg/mL, respectively, based on the previously obtained information [12]. Since we used IL for the first time on these cell lines, we tested a concentration range from 50 up to 200 µg/mL and found that no experimental points showed in vitro toxic effects. Thus, we used IL at 150 µg/mL for the following experiments.

### 3.2. RIPACUT^®^ Showed a Notable In Vitro Antioxidant Activity

**Figure 2:**
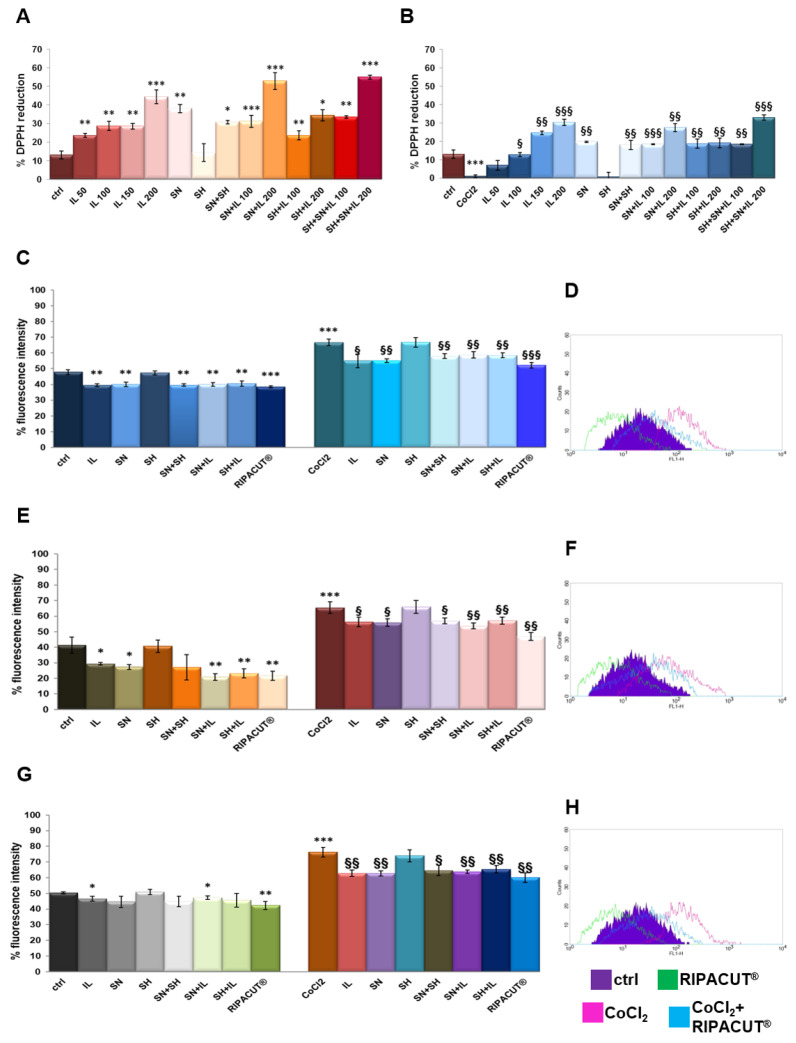
DPPH assay performed in a 96-well plate in the presence of IL (50 µg/mL, 100 µg/mL, 150 µg/mL and 200 µg/mL), SH (200 µg/mL), SN (30 µg/mL), SH + SN, 100 µg/mL IL + SN, 200 µg/mL IL + SN, 100 µg/mL IL + SH, 200 µg/mL IL + SH, 100 µg/mL IL + SH + SN and 200 µg/mL IL + SH + SN (**A**). The same assay was performed by adding 100 µM CoCl_2_ as a control of oxidation (**B**). DCF-DA assay on the HNEK (**C**), HNEpC (**E**) and FHC cells (**G**) treated or not for 24 h with 100 µM CoCl_2_ and 24 h more with IL (150 µg/mL), SA (200 µg/mL), SN (30 µg/mL), SH + SN, 150 µg/mL IL + SN, 150 µg/mL IL + SH and 150 µg/mL IL + SH + SN (RIPACUT^®^). In all the cases, representative flow histogram plots are reported in (**D**), (**F**) and (**H**) for the HNEK, HNepC and FHC cells, respectively, only for the cells treated or not with CoCl_2_ and then with RIPACUT^®^. The data represent the means ± SD. Note: * *p* < 0.05; ** *p* < 0.01; *** *p* < 0.001 vs. control; § *p* < 0.05; §§ *p* < 0.01; §§§ *p* < 0.001 vs. CoCl_2_.

Each single compound and a mixture of them obtained as different kinds of combinations were tested in order to evaluate potential antioxidant effects. For that reason, we performed a DPPH assay [19]. The assay was performed in the presence or absence of 100 µM cobalt chloride (CoCl_2_) since it is a well-known oxidizing stimulus. The results in Figure 2A show the marked antioxidant power of Iceland lichen extract at all the tested concentrations, with a major activity at 200 µg/mL. Silver nitrate also showed the ability to reduce the DPPH reagent, although less intense if compared to the lichen. These substances mixed together retained a higher antioxidant power, even if not significantly, as compared to their administration as single substances. The same tendency was shown in the presence of CoCl_2_ whose oxidant activity was strongly inhibited by IL starting from 100 µg/mL and the silver compound, both alone and in combination (Figure 2B). In no case sodium hyaluronate had any effect. Furthermore, through the DPPH assay, the EC_50_ for the lichen and the silver salt, as the only compounds showing antioxidant effects, was calculated, 318.57 µg/mL for the lichen extract and 59.88 µg/mL for the silver compound.

Then, the antioxidant effect on the HNEK, HNEpC and FHC cells was determined by a DCF-DA assay, a fluorescent probe whose signal increases in a directly proportional manner with the amount of reactive oxygen species. As reported in [12], the cell lines were incubated for 15 min after the selected treatments for 24 h either alone or after 24 h of pretreatment with 100 µM CoCl_2_. In Figure 2C, the graph reports the results of the fluorescence signal obtained on the HNEK cells for all the experimental points. On the other hand, the plot is representative of the fluorescence profile of RIPACUT^®^ in the presence or absence of CoCl_2_ against the relative controls as shown in the legend (Figure 2D). In parallel, Figure 2E,G shows the histogram reporting the percentage of the DCF fluorescence intensity evaluated with flow cytometry on the HNepC and FHC cell lines. The representative profiles in Figure 2F,H further prove the effects of RIPACUT^®^ with or without CoCl_2_ in the same cells. On all the three cell lines assessed, 150 µg/mL IL and 30 µg/mL SN maintained an antioxidant action (only on the FHC cells, silver nitrate had no notable effect in the absence of an oxidant agent), also preserving from the oxidative insult of CoCl_2_. Together, IL and SN showed a slightly increased antioxidant activity, even if not significantly, in comparison with their single administration. Although sodium hyaluronate showed a protective effect in no case, its combination with the other two substances reached a more intense antioxidant effect. In particular, the analysis of this combination in human keratinocytes reported a significantly reduced level compared to all the other experimental points (Figure 2C,D). Beside the interesting tendency we observed, this reduction was more marked in comparison with the other cell lines (Figure 2E,F for the HNEpC cells and Figure 2G,H for the FHC cells). Furthermore, in no case a single compound negatively affected the other ones in all the different kinds of double or triple combinations. Generally, with RIPACUT^®^, the intensity of fluorescence of the cells decreased significantly with respect to treatment with CoCl_2_ and also to untreated cells.

### 3.3. Iceland Lichen Extract Showed no Antibacterial Effects

**Figure 3:**
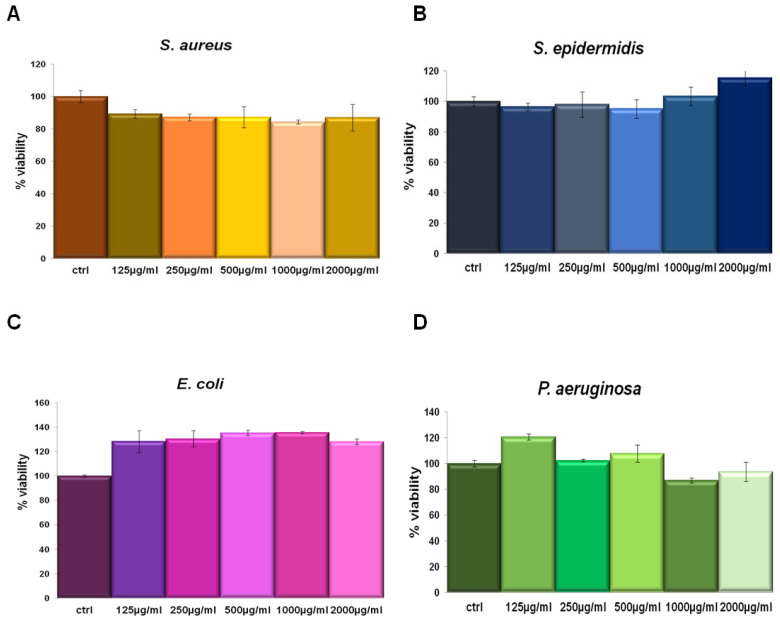
*S. aureus* (**A**), *S. epidermidis* (**B**), *E. coli* (**C**) and *P. aeruginosa* (**D**) incubated for 24 h with 125 µg/mL, 250 µg/mL, 500 µg/mL, 1000 µg/mL and 2000 µg/mL IL.

IL was tested in a concentration-dependent manner from 125 µg/mL to 2000 µg/mL against *Staphylococcus aureus* and *Staphylococcus epidermidis* (as Gram-positive) and *Escherichia coli* and *Pseudomonas aeruginosa* (as Gram-negative) bacterial strains. This assay was performed since we had no information about the potential activity of this compound on bacterial viability. In none of the tested concentrations the extract affected cell viability (Figure 3). Thus, we could utilize IL at 150 µg/mL for the following experiments on the same bacterial strains keeping the same concentration also used on human cell lines.

### 3.4. The Notable Antimicrobial Activity of RIPACUT^®^ Is Due to the Silver Compound

**Figure 4:**
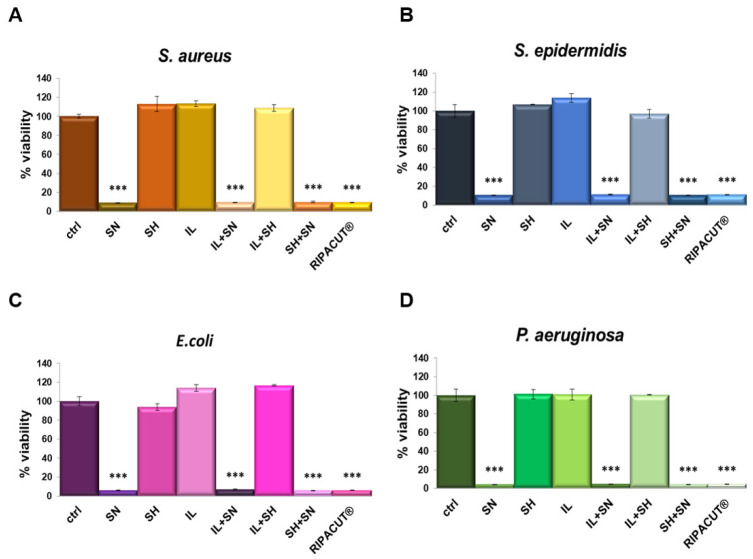
*S. aureus* (**A**), *S. epidermidis* (**B**), *E. coli* (**C**) and *P. aeruginosa* (**D**) incubated for 24 h with the indicated test solutions: IL (150 µg/mL), SN (30 µg/mL), SH (200 µg/mL), IL + SN, IL + SH, SH + SN, IL + SH + SN (RIPACUT^®^). Post-incubation, the bacterial cultures were measured at 600 nm and the results are expressed as the means ± SD. Note: *** *p* < 0.001, the data were analyzed by means of Student’s *t*-test.

The antibacterial activity of each solution was determined against the four bacterial strains previously evaluated (the Gram-positive *Staphylococcus aureus* and *Staphylococcus epidermidis* and the Gram-negative *Escherichia coli* and *Pseudomonas aeruginosa)*. As reported in Figure 4, the most notable antimicrobial effect was revealed only with silver, alone and in combination. Moreover, its antimicrobial activity was comparable to all the assessed conditions, suggesting that silver also retains its efficacy in combination with the indicated compounds. In this way, we further showed that the mixture with the other compounds, sodium hyaluronate and the extract of Iceland lichen, did not negatively affect the significant antibacterial effects of silver nitrate. As reported for the other assay, in this case, the other substances did not interfere with the effects of the silver salt as well.

### 3.5. RIPACUT^®^ Mediates Anti-Inflammatory Effects by Inhibiting the Release of the Main Cytokines

**Figure 5:**
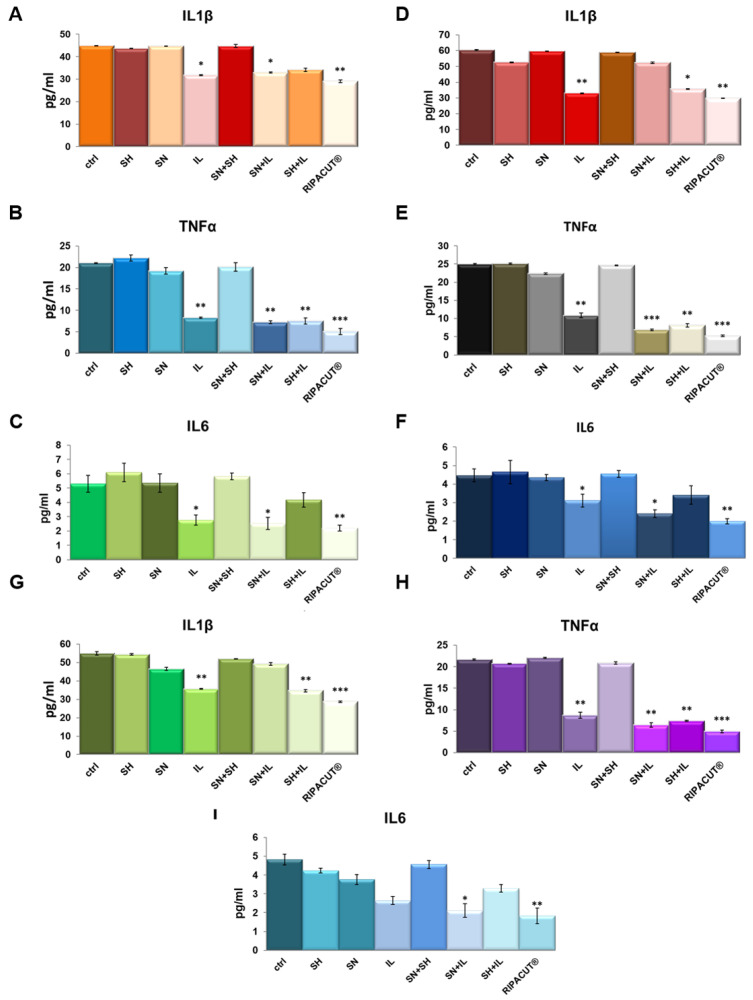
Evaluation of IL-1β (**A**), TNF-α (**B**) and IL-6 (**C**) production in the HNEK supernatants. The same cytokines were analyzed on the HNePC (IL-1β (**D**), TNF-α (**E**) and IL6 (**F**)) and FHC cells (IL-1β (**G**), TNF-α (**H**) and IL-6 (**I**)). The analyses were performed by means of an ELISA kit. The cells were treated for 24 h with IL (150 µg/mL), SN (30 µg/mL), SH (200 µg/mL), IL + SN, IL + SH, SH + SN, IL + SH + SN (RIPACUT^®^). The data represent the means of three independent experiments with similar results ± SD and were analyzed by means of Student’s *t*-test. Note: * *p* < 0.05; ** *p* < 0.01; *** *p* < 0.001.

In order to test potential anti-inflammatory effects of the combination of our interest, we performed ELISA assays on the supernatants of the HNEK, HNepC and FHC cells harvested after the treatments with Iceland lichen extract, the silver salt and sodium hyaluronate alone and differently mixed together. We evaluated IL-1β, TNF-α and IL-6 as the most characterized proinflammatory cytokines [20,21]. Here, we show that in basal conditions, Iceland lichen extract was able to significantly reduce the amount of all the cytokines we tested from the three cell lines. In the HNEK cells, this substance showed a more manifested effect in the decrease of TNF-α (Figure 5B). In the HNepC cells, it also showed a notable effect on IL-1β beside TNF-α (Figure 5D,E). Finally, the same tendency was highlighted for the FHC cells for which both cytokines were subjected to a stronger reduction (Figure 5G,H). The most interesting finding focused on the combination of the lichen extract with the other substances of our interest. In this case, on the keratinocytes, the whole combination appeared capable to inhibit the secretion of IL-1β, TNF-α and IL-6 (Figure 5A–C). Next, the prominent result on the nasal epithelial cells concerned the whole mix for IL-6 (Figure 5F) and TNF-α whose secretion was also decreased by mixing IL with only silver nitrate (Figure 5E). On the other hand, IL-1β did not show any changes in the presence of the complete combination as compared to the single IL administration. Finally, for the three cytokines on the FHC cells, the complete combination was able to reduce their levels in a more significant manner in comparison to the other experimental points including IL addition (Figure 5G–I).

### 3.6. RIPACUT^®^ Is Able to Revert the In Vitro Proinflammatory Effects of LPS

**Figure 6:**
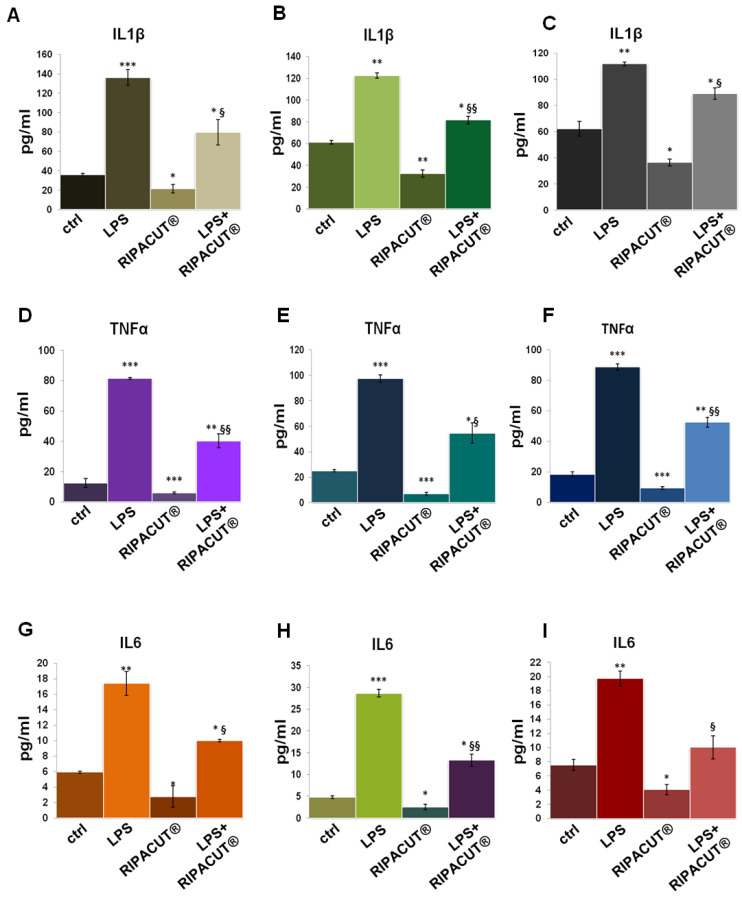
Evaluation by means of an ELISA assay of IL-1β (**A**), TNF-α (**D**) and IL-6 (**G**) production in the HNEK supernatants; IL-1β (**B**), TNF-α (**E**) and IL-6 (**H**) in the HNepC medium; IL-1β (**C**), TNF-α (**F**) and IL-6 (**I**) on the FHC cells. The cells were treated for 24 h with 1 µg/mL LPS and RIPACUT^®^. The data represent the means of three independent experiments with similar results ± SD. Note: * *p* < 0.05; ** *p* < 0.01; *** *p* < 0.001 vs. control; § *p* < 0.05; §§ *p* < 0.01; vs. LPS treatment.

ELISA assays were further performed on the HNEK, HNepC and FHC supernatants after the administration of LPS with and without RIPACUT^®^. As shown in Figure 6, in all cases, the mixture of our interest featured a notable ability to revert the inflammatory stimulus provoked by the endotoxin. The levels of IL-1β (Figure 6A for the HNEK, B for HNepC and C for FHC cells), TNF-α (Figure 6D for the HNEK, E for HNepC and F for FHC cells) and IL-6 (Figure 6G for the HNEK, H for HNepC and I for FHC cells) appeared strongly enhanced following LPS treatments. Interestingly, in the case of coadministration with the combination, the release of these cytokines was reduced in a significant manner in comparison to the administration of LPS alone.

## 4. Discussion

Intrinsic factors, such as unhealthy nutrition, diabetes or vascular diseases, as well as extrinsic ones such as falls and other accidents, pressure damage, and surgical operations are known to alter the integrity of epithelial barriers. Addressing these factors requires efforts from patients and clinicians in order to determine the appropriate treatment [22]. The development of new pharmacological and/or cosmetic approaches comprising natural products has acquired great importance, mainly focusing on topical formulations [23,24,25].

In this scenario, this work had the purpose of characterizing a combination of these three substances, named RIPACUT^®^, by focusing on several kinds of biological roles. Specifically, redox reactions strongly affect the fate of cells in healthy and pathological conditions [26]; for that reason, we first assessed the antioxidant activity of this combination. In this case, the outcome of the strong antioxidant effect due to the extract of Iceland lichen proved here on human keratinocytes, nasal and intestinal epithelial cells represents the first encouraging pharmacological aim. More interestingly, this antioxidant effect induced by Iceland lichen is also related to the silver salt. Thus, this in vitro antioxidant activity is assessed as notably improved when the substances are mixed together. Furthermore, the used concentrations of the lichen and the silver salt as the only antioxidant agents were notably lower than the assessed EC_50_. This represents an important aspect in the achievement of pharmacological enhancement obtained with their combination in comparison to the single treatments. All these features are supported by the use of cobalt chloride which added information about the ability of the combination to strongly improve the cell response under a stress condition. Indeed, this agent is known to mimic a hypoxia environment, a situation known to be responsible for the production of oxygen radicals and their following cell toxicity [27,28]. The partial reversion of the oxidation status on the three cell lines assessed further proves the positive action of RIPACUT^®^. Nevertheless, more experiments have to be performed in this context to continue the evaluation of the oxidation degree on cells in the presence or absence of the analyzed compounds and of all their combinations, including following oxidative stimuli. In fact, the DCFH-DA probe, although it is widely utilized for detecting intracellular oxidative stress, has several limitations for such measurements. Among them, it is important to keep in mind that DCF could be oxidized by several one-electron-oxidizing species; its intermediate radical rapidly reacts with oxygen to form a superoxide; the increase in DCF fluorescence can be altered during apoptosis directly or indirectly mediated by cytochrome c; other redox-active metals could interfere with the DCF activity [29].

Addition of the silver salt was due to the strong antimicrobial action which, in unhealthy conditions of skin and mucosa, can notably help to prevent or counteract the formation of an invasive microbiome causing infections [30]. Indeed, in order to preserve the good homeostasis balance of skin and mucosa, in case of limited superficial infections, a topical treatment represents an alternative to the use of systemic antibiotics and should be considered [31]. In this context, silver has a long history of use in human healthcare and medicine so much so that advances in medical-grade silver technology in the form of safer and bioavailable silver compounds and new delivery techniques have been developed in the past decades [32]. Interestingly, the most recent use of silver salts regards their encapsulation in nanoparticles which are nowadays known to be used in a plethora of biological activities. For instance, nano-silver formulations find application in the treatment of wounds, burns, in water disinfection systems, in the development of nano-containing materials for bone and dental implants besides the most common antibacterial, antiviral, antiprotozoal, antiarthropod and antitumor agents [33]. Moreover, recent case studies have reported the susceptibility of patients to the potential negative effects of these silver-containing products [34]. Our finding of the ability of RIPACUT^®^ to interfere with the production of proinflammatory cytokines has allowed us to consider it as an appealing protective opportunity as early as in the preliminary phase. Indeed, the key element of this combination is represented by the extract of Iceland lichen which has been historically used as an external remedy for vaginal discharge, boils and wounds [35]. Thus, in addition to its antioxidant capacity, which in turn is attributable to the content of phenols and their ability to scavenge free radicals [3], its therapeutic effectiveness also focuses on the ability to change the cytokine profile by shifting the balance towards the anti-inflammatory action. In our case, the inhibition of the release of such cytokines as IL-1β, TNF-α and IL-6 from all the epithelial cell lines notably induced by Iceland moss is in accordance with the previous outcomes regarding its use in the treatment of the oral mucosa inflammation occurring after nasal surgery or intubation and for simple infections of the throat. Particularly, in this model, it has been found that the aqueous extract of Iceland lichen causes an augmented secretion of IL-10 and IL-12p40 as anti-inflammatory cytokines [36]. Moreover, this compound is also most commonly used to alleviate colds, coughs, mouth irritation, and respiratory issues because, among other things, it is associated with the absence of side effects or interactions, and has also been studied for the treatment [37]. As assessed regarding the interference with the cell oxidation status, the marked decrease of cytokine release induced by the complete combination, including in the case of LPS proinflammatory stimulus, following a still unknown mechanism makes RIPACUT^®^ highly deserving of scientific interest for future experiments of molecular characterization. In this way, we propose studying the interaction of the used compounds not only as in vitro evaluation, but also deepening the knowledge in more complex systems.

Generally, topical treatment of different types of injuries has to present film-forming and hydrating qualities [38]. In our study, the addition of sodium hyaluronate, which apparently does not retain any biological activity in the analyzed processes, was considered to help obtain useful features in terms of hydrophilicity and wettability and positively affect the mechanical properties of pharmaceutical or even cosmetic formulations.

It is important to underline that the topical formulation considered for RIPACUT^®^ allows disregarding an in-depth series of analyses about its potential systemic side effects and/or interactions between molecules. Indeed, focusing on a single component, most of the previous studies were conducted on the lichen as whole extracts, and there is still little information about the derivative isolated compounds. This aspect represents an important limit for the complex of knowledge about this natural substance with growing pharmacological and, in general, biological relevance. Moreover, lots of evaluations have been carried out in vitro, and few in vivo, with no clinical trials using *Cetraria* species to evaluate safety, efficacy and toxicity. Thus, alongside the better-defined beneficial action, such side effects as itching, nausea, abdominal pain, heartburn, and burning if adsorbed per os for 2 weeks have been reported in fewer than 1% of the cases [39,40]. Side effects could be also due to the ability of the lichen to adsorb heavy metals from polluted air. Interestingly, no known drug interactions have been reported for Iceland lichen even though it contains fibrous mucilage compounds which may decrease the absorption of medications and reduce their efficacy [40]. Similar aspects could be considered for the other two substances we tested in this work. In particular, the silver salt is known to provoke signs of an allergic reaction besides other minor effects also due to the topical administration, described as a change in color of the skin and irritation where this drug is used [41]. The possible interactions of the silver salt with other substances are reported with some kinds of anti-inflammatory molecules, penicillins and several kinds of electrolytes. Indeed, therapy with silver nitrate dressings should be applied cautiously in patients with electrolyte alterations. Finally, topical application of sodium hyaluronate could allow the appearance of skin allergic reactions, while no interactions have been described with the most common compounds used topically [42]. Definitively, all this information suggests that a favorable risk–benefit profile is supported for Iceland lichen extract, silver salt and sodium hyaluronate as single agents and also as a complete combination.

## 5. Conclusions

The data presented in this work allowed obtaining attractive preliminary evidence about RIPACUT^®^ as a combination of Iceland lichen extract, silver salt and sodium hyaluronate for the protection of skin and the epithelial barriers of nasal and anal areas. Interestingly, two components included in this combination showed specific effects regarding antioxidant, antimicrobial and anti-inflammatory activity. To these compounds, the presence of sodium hyaluronate represents an attractive expedient to further obtain the barrier effect as a film-forming polymer also capable of keeping healthy moisture levels. Thus, the choice of these three substances covers three different actions in a targeted manner, with both biological relevance and concerning the pharmacological formulation. The conception of this kind of product allows us to consider the in vitro analysis we carried out here as an interesting starting point to elaborate further assessment in the form of in vivo experiments. These tests can be a useful means of validating RIPACUT^®^ as an active principle of a new topical formulation for skin, nasal and anal mucosa. In this study, we addressed a pharmacological development which could actively act as a protective agent against oxidative, inflammatory and infective insults as the main causes of the alteration of the epithelial homeostasis.

## Data Availability

The data can be shared up on request.
